# Phytoplankton bloom stages estimated from chlorophyll pigment proportions suggest delayed summer production in low sea ice years in the northern Bering Sea

**DOI:** 10.1371/journal.pone.0267586

**Published:** 2022-07-08

**Authors:** Clare B. Gaffey, Karen E. Frey, Lee W. Cooper, Jacqueline M. Grebmeier

**Affiliations:** 1 Graduate School of Geography, Clark University, Worcester, Massachusetts, United States of America; 2 University of Maryland Center for Environmental Science, Solomons, Maryland, United States of America; Institute of Oceanology Chinese Academy of Sciences, CHINA

## Abstract

Decreased sea ice cover in the northern Bering Sea has altered annual phytoplankton phenology owing to an expansion of open water duration and its impact on ocean stratification. Limitations of satellite remote sensing such as the inability to detect bloom activity throughout the water column, under ice, and in cloudy conditions dictate the need for shipboard based measurements to provide more information on bloom dynamics. In this study, we adapted remote sensing land cover classification techniques to provide a new means to determine bloom stage from shipboard samples. Specifically, we used multiyear satellite time series of chlorophyll *a* to determine whether in-situ blooms were actively growing or mature (i.e., past-peak) at the time of field sampling. Field observations of chlorophyll *a* and pheophytin (degraded and oxidized chlorophyll products) were used to calculate pheophytin proportions, i.e., (Pheophytin/(Chlorophyll *a* + Pheophytin)) and empirically determine whether the bloom was growing or mature based on remotely sensed bloom stages. Data collected at 13 north Bering Sea stations each July from 2013–2019 supported a pheophytin proportion of 28% as the best empirical threshold to distinguish a growing vs. mature bloom stage. One outcome was that low vs. high sea ice years resulted in significantly different pheophytin proportions in July; in years with low winter-to-spring ice, more blooms with growing status were observed, compared to later stage, more mature blooms following springs with abundant seasonal sea ice. The detection of growing blooms in July following low ice years suggests that changes in the timing of the spring bloom triggers cascading effects on mid-summer production.

## Introduction

An unprecedented lack of winter sea ice led to open water conditions throughout the winter of 2017–2018 in the northern Bering Sea [1]. Sea ice extent in the Bering Sea in 2017–2018 was far lower than any previous winter in the reconstructed or observed past dating back to 1850 [2]. Unusual environmental conditions that led to the drastic sea ice reduction included residual heat from 2017 that delayed freeze-up in the Chukchi Sea, which then delayed ice production in the Bering Sea. Additionally, a large, persistent high-pressure system over the Aleutian Islands and southern Bering Sea from February through May 2018 shifted the position of the Aleutian Low Pressure System northwest over eastern Siberia, resulting in persistent southerly winds transporting warm air over the Bering Sea that prevented sea ice formation until March [1, 3]. At least in the short term, this event appears anomalous rather than a trend as the 2020 and 2021 period had sea ice recovering to more typical extents [4].

Sea ice is an important controlling factor for many of the biological characteristics observed on the Bering Sea shelf [5]. Declines in Pacific Arctic Region sea ice impact species at multiple trophic levels as well as overall ecosystem function [6, 7]. Following the 2017–2018 event, the northern Bering Sea had weakened water column stratification, a delayed spring phytoplankton bloom, and the typical persistence of cold bottom water (<2°C), termed the cold pool that extends through the summer, was much diminished. The cold pool may serve as a refuge for the early age class of juvenile Walleye pollock [8, 9], and low seawater temperatures (<0°C) exclude the commercially fished, larger size classes of this species [10]. Cascading effects of the sea ice decline has led to lower abundances of large crustacean zooplankton (large copepods and euphausiids) in the summer and coincident die-offs of summer seabirds [8] as well as reductions in lipid content and overwinter survival of age-0 pollock [11] that prey on large zooplankton. These biological consequences are tied to the timing of phytoplankton growth, meaning that the extent and persistence of seasonal sea ice is a critical element for understanding the function of the marine ecosystem. Monitoring efforts such as the Distributed Biological Observatory (DBO) in the Pacific Arctic Region provide the opportunity to improve our understanding of how phytoplankton growth and its phenology play a role in ecosystem organization [12, 13].

While other studies have evaluated the impacts on phytoplankton phenology from sea ice cover reductions in the fall [14] and spring months [15], including blooms initiated prior to sea ice breakup [16, 17], our focus here is on productivity in the mid-summer, in July. Changes in the timing of sea ice break up and subsequent timing of spring blooms could have follow-on consequences into the summer and potentially affect ecosystem function. We use here existing ship-based DBO datasets collected during annual July field sampling of chlorophyll *a* biomass, and combine these data with comparable satellite observations to offer a potentially more synoptic identification of mid-summer bloom patterns.

Repeat chlorophyll *a* observations from satellites have routinely been used to monitor surface phytoplankton bloom phenology, e.g. [14]. However, optical satellite sensors are limited in that they cannot detect chlorophyll *a* deep within the water column and the collection of useful data are further hindered by consistent cloud cover that is characteristic of the Pacific Arctic Region. A remedy to these limitations is to use ship-sampled chlorophyll *a* pigments as indicators of phytoplankton bloom stage. The proportion of active chlorophyll *a* relative to its oxidized breakdown product, pheophytin, can be used to determine whether chlorophyll *a* biomass reflects sampling during the growing phase of a phytoplankton bloom or during its senescence. Chlorophyll *a* is converted into pheophytin during microbial degradation and grazing activities [18, 19]. Pheophytin proportions relative to combined live chlorophyll *a* and pheophytin pigments have been used to qualitatively determine areas of relatively active blooms in the Arctic [20, 21], but these studies have been limited in analyzing only individual cruises and have not attempted to derive a more quantitative approach to describe bloom stage. More typically, pheophytin collections in Arctic regions have been used as a diagnostic marker for phytoplankton grazing processes [22–24] or used in conjunction with other pigments to characterize phytoplankton community composition [25]. Empirical quantitative relationships between chlorophyll *a* and pheophytin for predicting in-situ bloom stage have not been developed. Therefore, our novel objective in this study was to determine a threshold value of pheophytin to total chlorophyll *a* that would provide an estimate of in-situ phytoplankton bloom stage. Specifically, our goals were the following: (a) Can the proportion of pheophytin relative to combined pheophytin and chlorophyll *a* be used to estimate phytoplankton bloom stage using satellite-measured time series; and (b) has there been any measurable impact on the July phytoplankton bloom phenology owing to earlier sea ice retreat or limited formation during the prior winter in the northern Bering Sea?

## Methods

### Remote sensing of surface phytoplankton phenology

Remotely sensed bloom stages were derived from sea surface chlorophyll *a* measurements, using a methodology that is an extension of common approaches for remote sensing land cover classification [26–29]. Typically, these studies involve comparisons of classifications derived from satellite remote sensing and “ground truthing” verified through local surveys or by using reference remote sensing sources with higher spatial resolutions [30]. Unlike these typical land cover classification applications, there is no available “ground truth” for our study due to the lack of continuous in-situ sampling that would be required to directly determine bloom stage. Therefore, to examine the relationship between remotely sensed phenology and field-based cruise measurements, satellite observations were considered the reference classification since stations are observed continuously over time.

**Fig 1:**
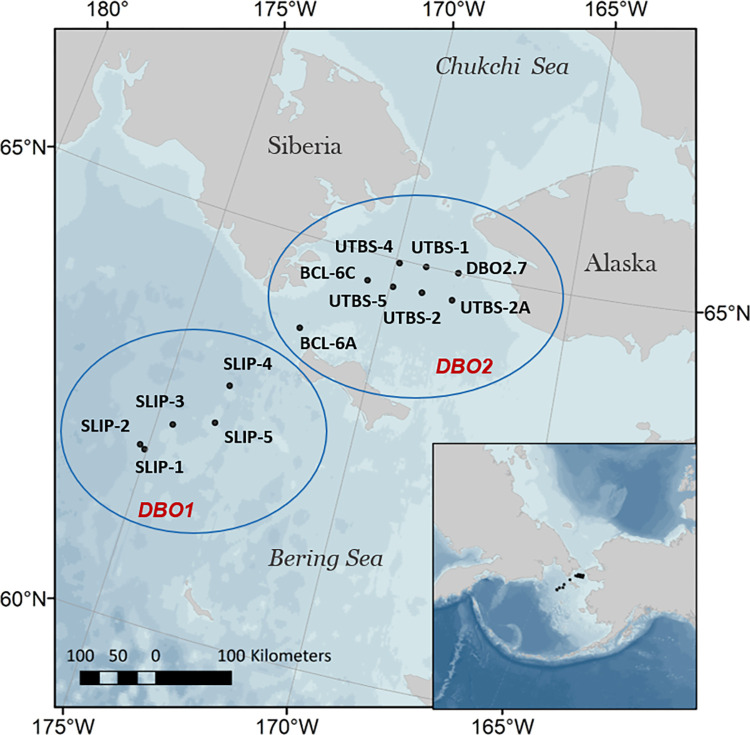
Map of the northern Bering Sea study area. Two DBO areas are indicated in red and the black text corresponds to individually sampled stations within each DBO transect marked by the blue circles. The stations are grouped as DBO1 (n = 5: SLIP-1, SLIP-2, SLIP-3, SLIP-4, SLIP-5) and DBO2 (n = 8: BCL-6A, BCL-6C, UTBS-1, UTBS-2, UTBS-3, UTBS-4, UTBS-5, DBO2.7). The bathymetry is reproduced from GEBCO Sheet G.08 compiled by R.L. Fisher of the Scripps Institution of Oceanography and extracted from the GEBCO Digital Atlas published by the British Oceanographic Data Centre on behalf of the IOC and IHO, 2003.

Satellite remotely sensed time series of ocean surface chlorophyll *a* concentrations were used to resolve bloom cycles in order to determine whether chlorophyll *a* was increasing or decreasing on the dates of field sampling. Ocean color imagery collected by NASA MODIS-Aqua mission (https://oceancolor.gsfc.nasa.gov/), sourced from Google Earth Engine datasets at 500 m resolution from 2013–2019 were used to analyze phytoplankton growth patterns observable at the seawater surface. Satellite images were collected at the DBO sites every 1–2 days but owing to frequent cloud cover, the average number of days of useable imagery was 27 observations per year. Years with fewer than 6 total satellite observations were not evaluated to ensure only representative phytoplankton life stages were documented at a given location, which is a standard applied in previous work [31]. Complete (usually secondary) remotely sensed surface chlorophyll *a* blooms at the DBO Bering Sea sites indicated an average difference of 20 days between bloom peak and return to baseline values. Therefore, in order to balance satellite imagery availability and resolvable bloom activity relevant to shipboard observations, the period of interest was set to be the dates of annual field sampling (Day of Year (DOY) 194–199) ± 20 days. The quality of the chlorophyll *a* time series as it relates to field data was assured by excluding years with fewer than 6 chlorophyll *a* concentration observations, and by using a minimum of one observation every 25 days and a minimum of three chlorophyll *a* concentration determinations over the extended period (DOY 174–219) overlapping the shipboard sampling dates. These criteria enabled the removal of incomplete bloom data immediately prior and post cruise sampling. Furthermore, to remove chlorophyll *a* concentration signals that may be affected by the presence of sea ice, daily sea ice concentration estimates were used to mask any satellite image pixels that had greater than 15% sea ice concentration. To examine the chlorophyll *a* variability for each of 13 northern Bering Sea stations between sites DBO1 (n = 5: SLIP-1, SLIP-2, SLIP-3, SLIP-4, SLIP-5) and DBO2 (n = 8: BCL-6A, BCL-6C, UTBS-1, UTBS-2, UTBS-3, UTBS-4, UTBS-5, DBO2.7) (Fig 1), a buffer radius of 5.5 km was used. This buffer was selected because it was small enough to adequately represent each station yet large enough to mitigate loss of observations owing to persistent cloud cover. The median chlorophyll *a* concentration was extracted at each station from near-daily MODIS imagery rather than the arithmetic mean to reduce the dominance by unrepresentative, outlier pixels [32]. Satellite extracted chlorophyll *a* data was determined for each station within each year were then filtered using a locally weighted scatterplot smoothing function to further reduce outliers following previous methodologies [14, 31]. Finally, a daily chlorophyll *a* time series for each station year (defined as each year of cruise data at each station within the DBO1 and DBO2 sites) was produced via linear interpolation between data points.

**Fig 2:**
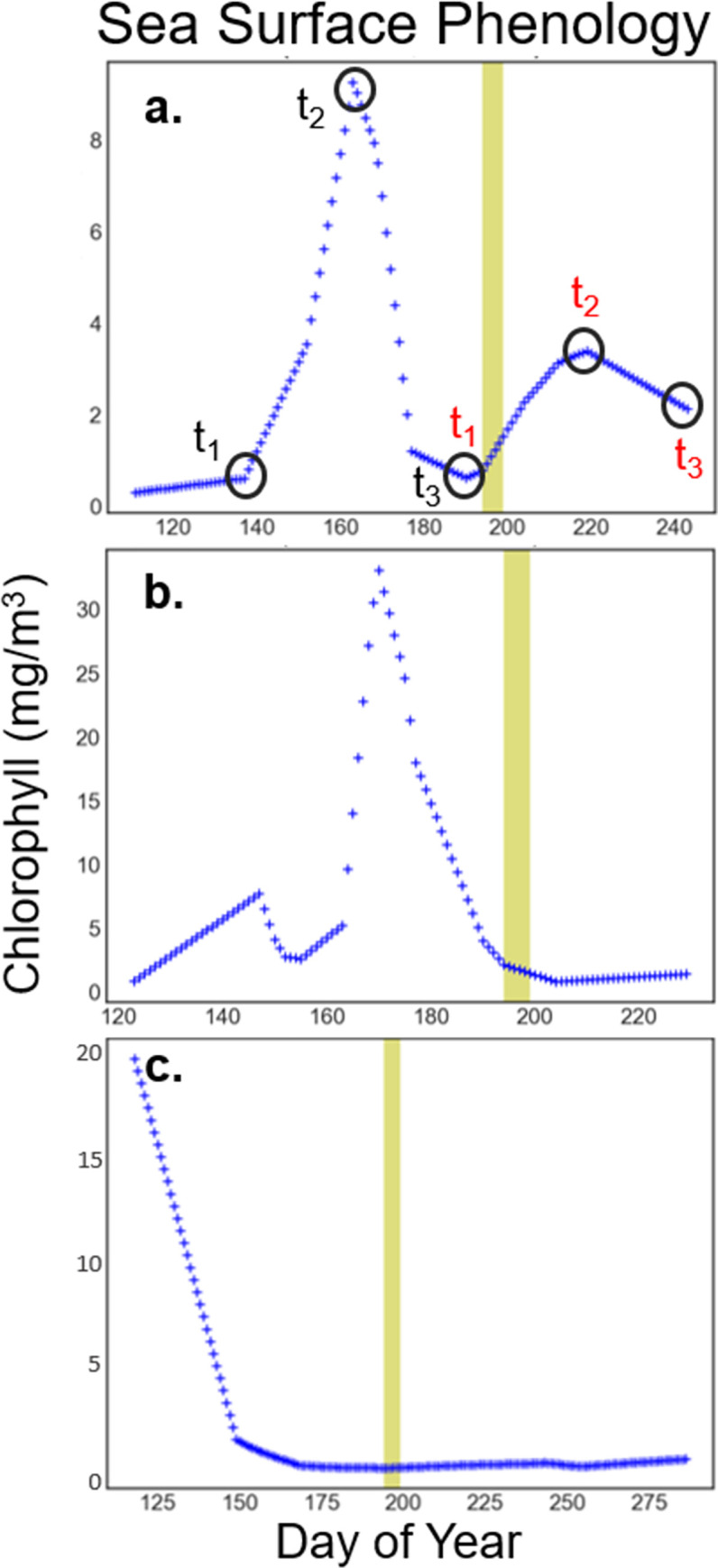
Examples of sea surface phytoplankton phenology curves using NASA MODIS Aqua chlorophyll *a* concentration. The examples listed are (a) DBO2 station UTBS-2 in 2013, (b) UTBS-4 in 2013, and (c) DBO1 station SLIP-1 in 2015. The dark circles and label indicate time 1, 2, and 3 (t_1_, t_2_, t_3,_ respectively) that were recorded for each bloom curve for the Day of Year (DOY) and chlorophyll *a* concentrations, at the day of year of the start, peak, and end of bloom (shown in plot (a) only). The metrics were recorded for each separate bloom (determined to be individual curves that rose at least 0.5 mg/m^3^ from its t_1_). Therefore, the second bloom pictured here was separately assigned t_1_, t_2_, and t_3_ in red. The yellow shaded bars represent the date range of all July cruise sampling 2013–2019 (DOY 194–199). The bloom stage was recorded for each bloom that corresponded to the time of cruise sampling. Therefore, the examples shown are (a) Early Bloom, (b) Post Bloom, and (c) No Bloom stages at the time of sampling.


Based on the remotely sensed phytoplankton growth curve at each DBO station, the life stage at time of sampling for each collection date 2013–2019 was estimated using a classification approach. We define these categories as No Blooms, Early Blooms (EB), and Post Blooms (PB) based upon the dates of start (*t*_1_), peak (*t*_2_), and end (*t*_3_) of bloom cycles within the phenology curves compared with the DOY of sampling (Fig 2). The start, peak, and end dates and associated chlorophyll *a* concentrations were recorded from each bloom if multiple blooms cycles occurred within a year at a single location (e.g. double bloom [14]). We used a threshold increase of 0.5 mg/m^3^ of surface chlorophyll *a* to identify additional individual blooms [32, 33]. Smaller increases were considered either continuations of a large bloom cycle or minor variability classified as No Bloom (Eq 1) depending on the context of each annual phenology curve. The timing of peak chlorophyll *a* concentrations is considered a focal identifier for describing phytoplankton phenology [34, 35]. To tease out within-bloom dynamics, EB and PB stages were distinguished based on sample dates preceding or following the chlorophyll *a* concentration peak within a bloom cycle respectively. Specifically, EB contained the section of the phenology curve from the start time (*t*_1_) until the maximum amplitude of chlorophyll *a* concentration at (*t*_2_) in Eq 2 and Fig 2. PB were considered to be any concentration that followed a specific time, t, *t*_2_ through *t*_3_ (Eq 3).

$$No\;Bloom\;DOY\;Sampled<t_{1}\;or\;DOY\;Sampled>t_{3}$$ (1)

$$Early\;Bloom\;t_{1}\leq DOY\;Sampled\leq t_{2}$$ (2)

$$\mathrm{Post}\;Bloom\;t_{2}<DOY\;Sampled\leq t_{3}$$ (3)

### Shipboard measurements

Seawater samples were collected on seven research cruises aboard the Canadian Coast Guard Ship (CCGS) *Sir Wilfrid Laurier* annually in July from 2013–2019 at DBO designated stations (Fig 1). DBO1 stations are located southwest of St. Lawrence Island and the DBO2 region is within the Chirikov Basin, which is north of St. Lawrence Island. Water was collected from the CTD rosette (Sea-Bird SBE25/33) using a SBE32 Carousel 12-bottle water sampler with 8-L bottles. Water samples were collected at set depth increments (i.e. 5, 15, 25, 35, 50, 75 m, bottom depth, and at the chlorophyll maximum). Chlorophyll *a* and pheophytin collection methods followed National Estuarine Research Reserve System Centralized Data Management Office protocols [36]. Briefly, seawater samples (200 mL) were immediately filtered on 25 mm Whatman GF/F filters in the dark. Filters were frozen shipboard (-20°C) and analyzed within three months post-cruise at Clark University. Chlorophyll *a* was extracted from filters using 90% acetone, and vials with acetone and filters were stored wrapped in foil in a freezer for at least 48 hours prior to measurement on a calibrated Trilogy Fluorometer (Turner Designs, San Jose, California). Pheophytin was determined following acidification of the samples. Cruise data are available at https://arcticdata.io/catalog/portals/DBO/Data.

### Pheophytin threshold model

Estimates of the life stage of the in-situ sampled bloom were determined using the proportion of pheophytin in the combined surface chlorophyll *a* and pheophytin concentrations [Pheophytin/(Chlorophyll + Pheophytin)] [21] of field analyzed surface (5 m) samples. Pheophytin proportion thresholds were assessed empirically to classify stages of phytoplankton bloom cycles (EB versus PB) in accordance with satellite-derived phenology. Specifically, using the full 2013–2019 DBO1 and DBO2 dataset, consecutive integer pheophytin percentage thresholds (0–100%) were incrementally compared to corresponding satellite-derived EB and PB classes to select the threshold that best matched the satellite classes. The threshold testing followed the logic that a higher percentage of pheophytin indicated a mature PB while samples deplete of pheophytin represented growing EB. The final threshold selection was based on which model produced the maximum overall accuracy and minimized classification bias determined using omission and commission errors. These were calculated by reviewing the incorrect classifications of the reference (remotely sensed classes) and classified model (pheophytin proportions) for each class type following traditional remote sensing classification techniques [37]. The No Bloom class was not considered in the analysis because of the challenges of distinguishing whether a No Bloom period contained residual chlorophyll *a* from a previous bloom or if it followed a more prolonged unproductive state.

### Sea ice impacts on in-situ chlorophyll *a* pigments

The impact of spring sea ice on mid-summer phytoplankton phenology was analyzed for each station year based on high and low ice years, which were defined by sea ice breakup dates and daily sea ice concentrations. Springtime sea ice concentrations were considered in addition to sea ice breakup dates to account for instances in which ice recovered and limited light penetration into the sea surface which may have obstructed photosynthesis and therefore bloom growth. Daily sea ice data were obtained from the Special Sensor Microwave/Imager (SSM/I) and Special Sensor Microwave Imager/Sounder (SSMIS) passive microwave instruments carried on Defense Meteorological Satellite Program satellites. Sea ice concentration estimates were calculated using the Goddard Bootstrap (SB2) algorithm [38, 39] at 25 km spatial resolution. Sea ice breakup dates were determined following methodology in [4, 40]. Briefly, breakup date each year was defined using satellite imagery when a 25 km pixel observed two consecutive days below a 15% sea ice concentration threshold. A change point analysis using the method At Most One Change (AMOC) [41, 42] was conducted to determine a DOY threshold parameter to distinguish high versus low sea ice years according to the sea ice breakup date specific to each station [43]. The change point method ensured a representative threshold would be selected for each study site along the latitudinal gradient. Station years were categorized as low or high sea ice years based on whether the sea ice breakup occurred before or after the DOY threshold, respectively (see details in S1 Appendix). In addition to sea ice breakup dates, high and low sea ice years were categorized directly by sea ice concentrations during the late winter-spring transition (March 1–May 1), when increasing daylight would activate spring blooms. High ice years were considered the default while any year with zero percent sea ice concentrations for more than five consecutive days in the March 1–May 1 timeframe was classified as a low ice year.

Pheophytin proportions determined from water column field samples as described above were grouped based on high and low sea ice years to investigate the impact of low sea ice on subsequent bloom phases in July. Depth-integrated pheophytin proportions were considered in addition to surface samples due to decreased sensitivity to wind-driven mixing and rapid nutrient depletion at depth compared to surface chlorophyll *a* concentrations. Therefore, surface (5 m) and depth-integrated chlorophyll *a* and pheophytin concentrations measured at each station were used to investigate the impact of high and low sea ice years on pheophytin proportions throughout the water column. The samples were binned according to low and high ice years and Welch’s t-test was used to compare the two groups to determine if their averages were significantly different from one another. The statistics were performed using the “numpy” and “scipy” packages in the Python programming language (Python Software Foundation, https://www.python.org/) [44, 45].

## Results

### Chlorophyll *a* bloom stage classification

Nine remotely sensed time series curves were excluded due to not reaching the criteria for consistent observations (i.e. a minimum of one chlorophyll *a* value every 25 days and a minimum of three chlorophyll *a* values between DOY 174–219). The excluded data were from BCL-6A and all DBO1 stations in 2013, as well as SLIP-1, SLIP-2, and SLIP-3 in 2018 (see Fig 1 for station location). With the exclusion of these station years and the exclusion of the No Bloom remotely sensed classes, data from twenty-eight stations in the study years were available to compare between field and satellite classifications and to test the compatibility of the two methods for determining bloom stage (early (including growing) and post bloom). Based on this dataset, a 28% pheophytin proportion threshold relative to the combined surface chlorophyll *a* and pheophytin concentrations produced the highest overall accuracy for distinguishing EB and PB classes based on corresponding satellite-derived bloom stage classes. Therefore, samples that contained less than a 28% pheophytin proportion were classified as EB and considered to be growing. Samples with more than 28% pheophytin proportion were classified as PB, meaning they were sampled post peak according to the remotely sensed time series. An error matrix and accompanying accuracy assessment are available in Table 1. The 28% pheophytin proportion threshold provided an overall accuracy of 75% in distinguishing EB vs. PB according to the remotely sensed bloom staging criteria. Of the twenty-three satellite EB (used as a reference), the threshold model correctly classified sixteen samples, while the model correctly matched all five referenced PB samples.

**Table 1:** Field measured vs. remotely sensed life stage classification accuracy assessment of phytoplankton stage.

		Satellite					
		EB	PB	Total	N = 28	Consistent	Inconsistent
Field	EB	16	0	16	Number	21	7
	PB	7	5	12	Percentage (%)	75	25
	Total	23	5				

Error matrix (left panel) and accuracy results (right panel) of the developed field classification scheme against the remotely sensed classes of life stage at the time of cruise sampling using a pheophytin proportion of 28% for each field water column sample taken at 5 m depth. EB = Early Bloom, PB = Post Bloom. Note that only exact matches for station years with both satellite and field data available were used.

**Fig 3:**
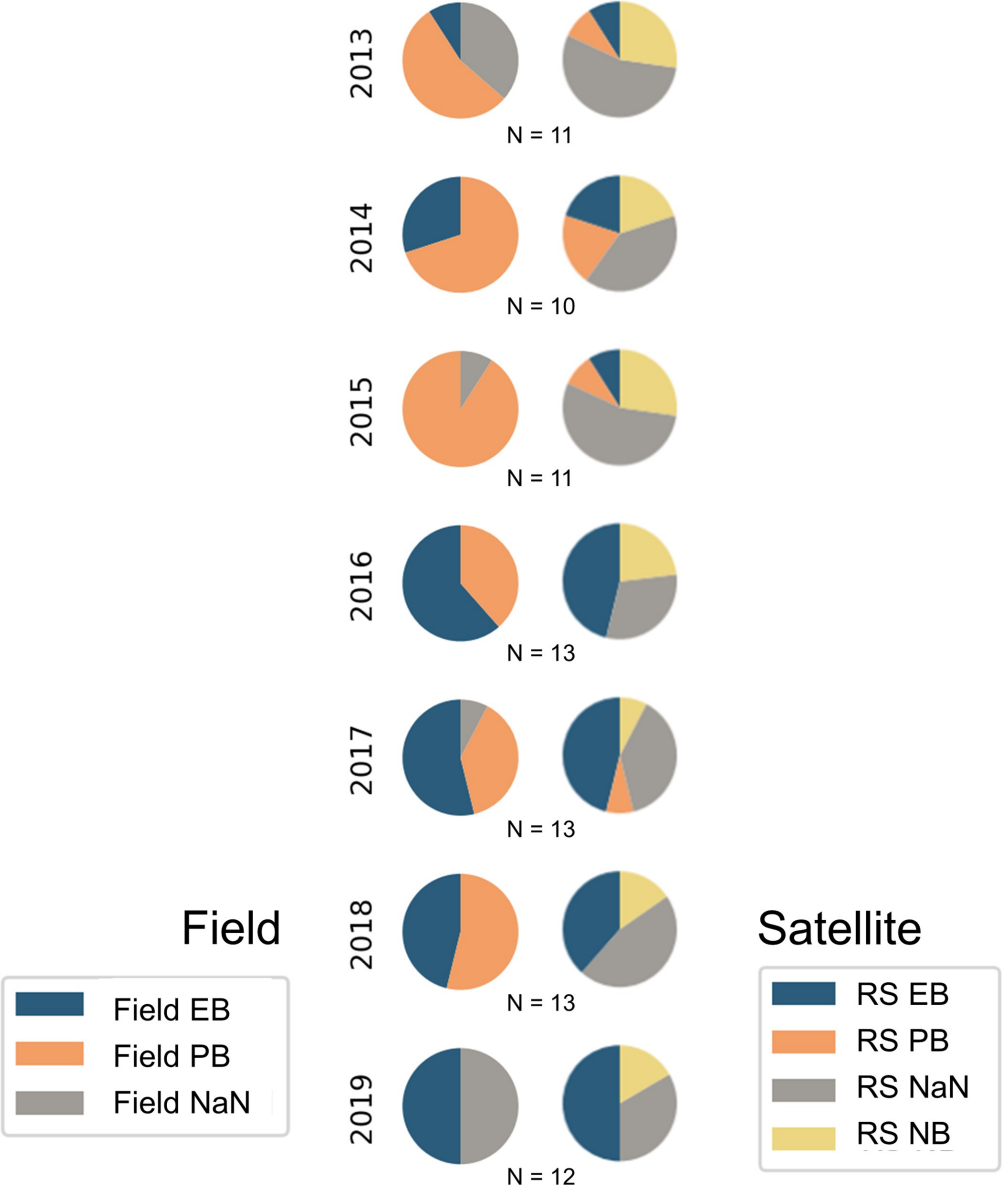
The aggregated counts of bloom stages identified for all of the stations within DBO1 and DBO2 for both the remotely sensed (RS) and cruise-collected (field) pheophytin proportion methods. EB = Early Bloom, PB = Post Bloom, NaN = missing data, NB = No Bloom for observations outside of a bloom cycle. The aggregation was performed using all available years of data (i.e. years that failed the minimum observations criteria for satellite time series are classified here as “RS NaN”).

Results for the field and satellite annually aggregated classes for all DBO1 and DBO2 stations are shown in Fig 3, and individual station matchups for each year are provided in S2 Table. The annually aggregated results (Fig 3) of all classified station years for both the remotely sensed and field collected datasets included instances of missing observations (indicated as NaN). The majority of the DBO1 stations did not have adequate remote sensing observations available to resolve surface phenology in years 2013–2015 and 2018, limiting data availability needed to compare the classification model performance of DBO1 and DBO2 separately. On the other hand, remotely sensed classifications were successfully produced for the majority of DBO2 stations each year.

### Sea ice and pheophytin proportions

**Fig 4:**
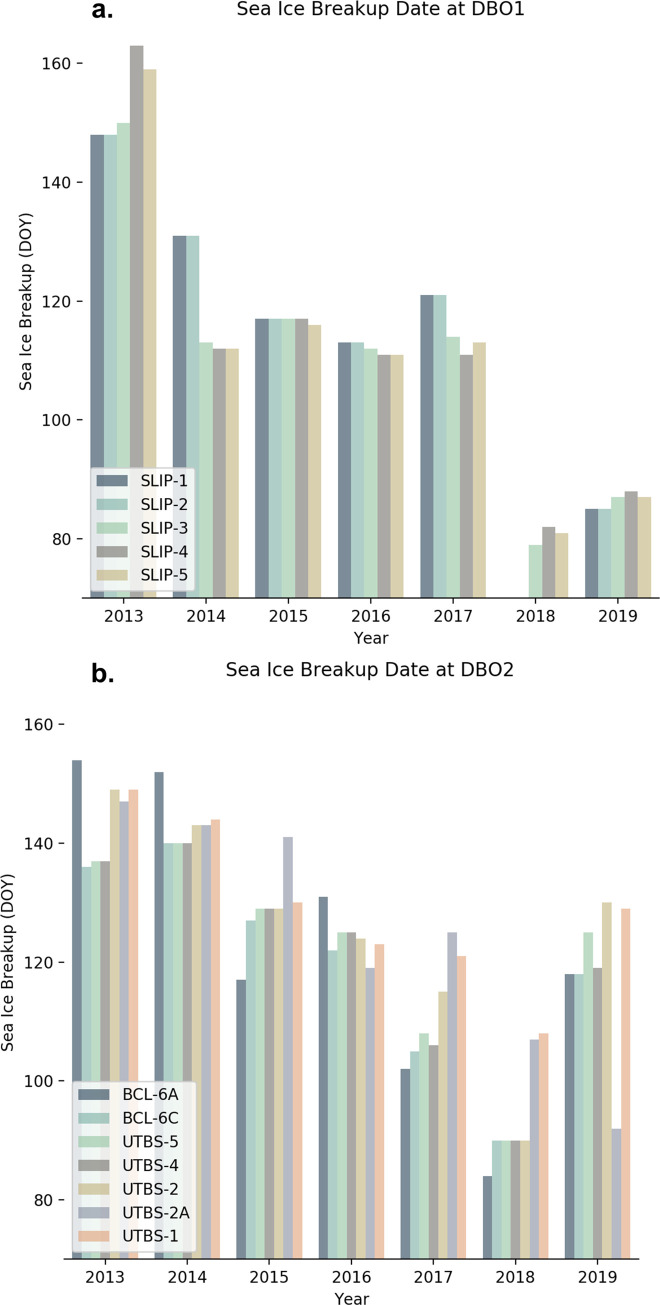
Sea ice breakup dates. Sea ice breakup dates for DBO1 (a) and DBO2 (b) over the time series. Bars are missing at some 2018 DBO1 stations because winter sea ice did not form and no breakup date was observed.

**Fig 5:**
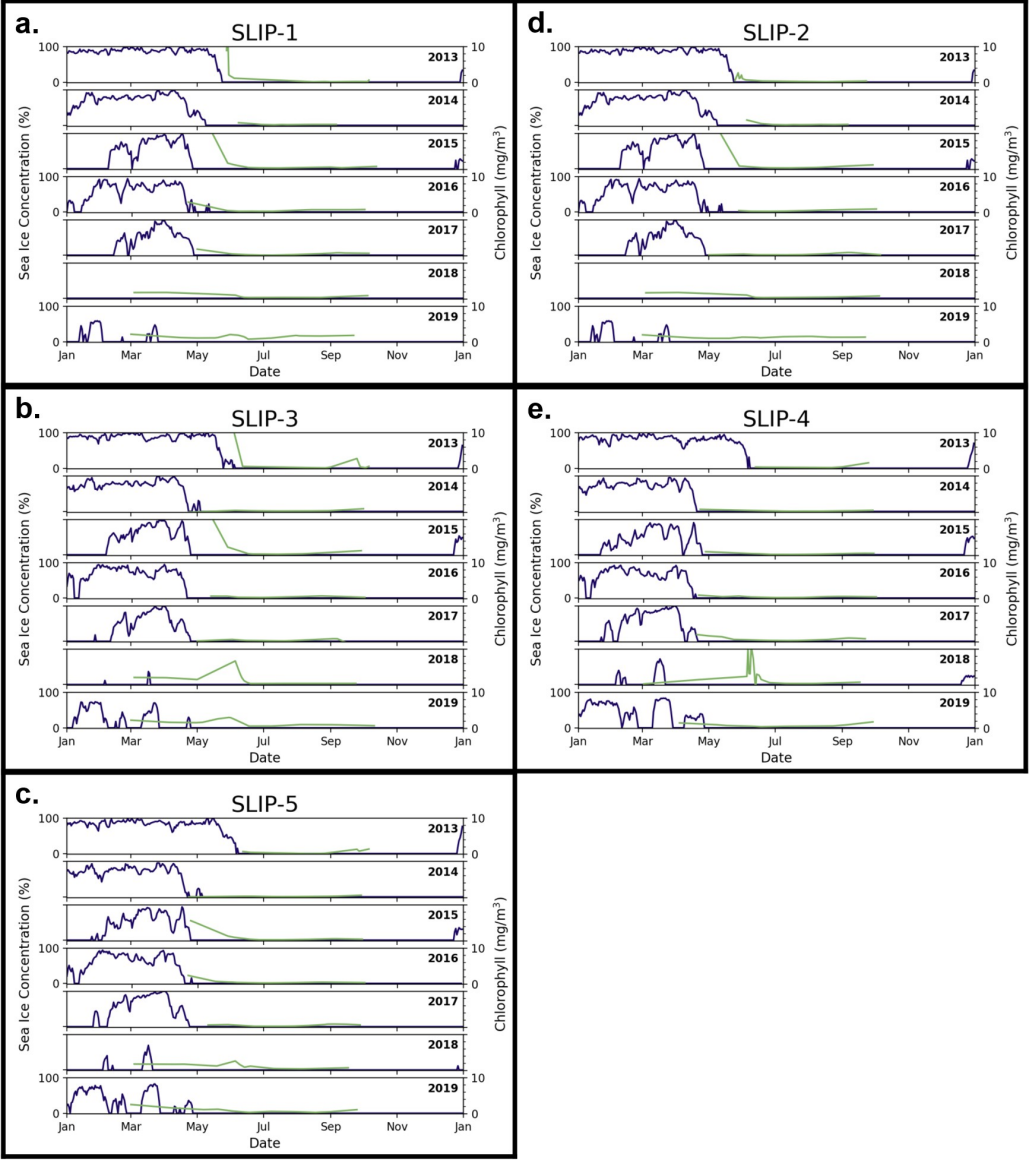
Daily sea ice concentrations (blue) and MODIS chlorophyll *a* (green) time series curves at the DBO1 stations.

**Fig 6:**
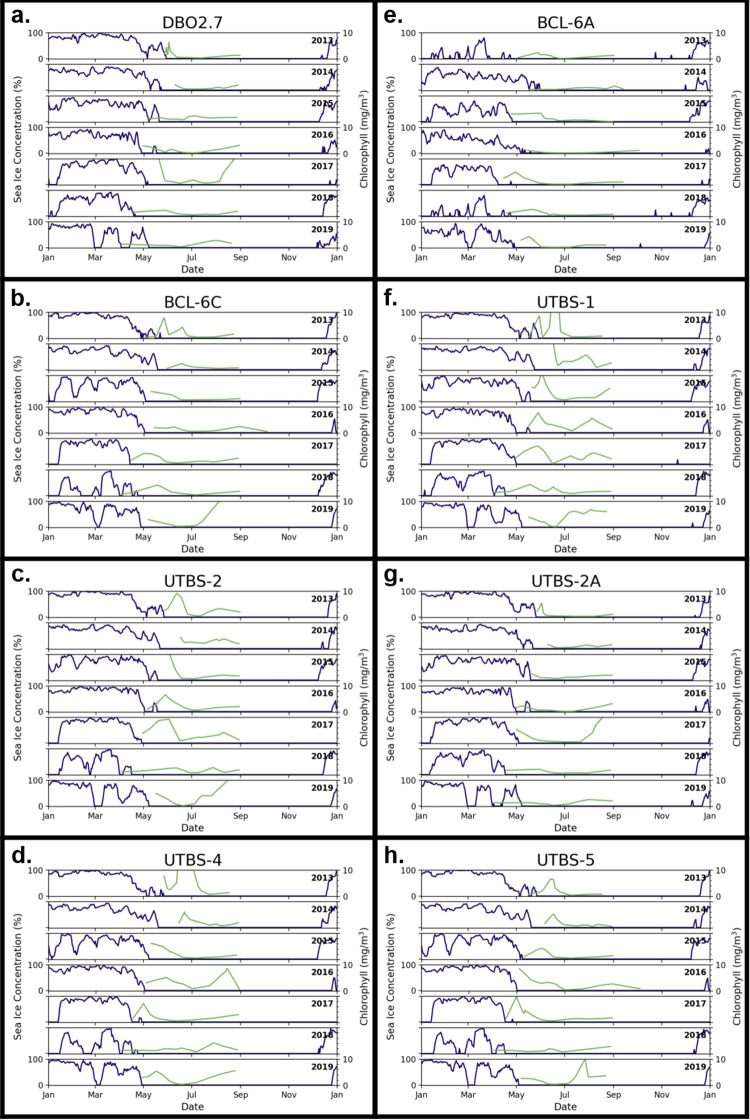
Daily sea ice concentrations (blue) and MODIS chlorophyll *a* (green) time series curves at the DBO2 stations.

High and low sea ice station years were determined using sea ice breakup date (Fig 4, S4 Table) and sea ice concentration (Figs 5 and 6, S4 Table). Interannual sea ice dynamics were generally similar throughout the Bering Sea stations, although some distinctions between DBO1 and DBO2 were evident. In 2018, no sea ice breakup nor concentration data were presented because winter sea ice did not form at stations SLIP-1 and SLIP-2 (Figs 4(A), 5(A) and 5(D)). The remaining stations in DBO1 had continuous ice cover for less than a month late in the season. At the same time, DBO2 had reduced sea ice cover with periods of open water in spring (March 1–May 1), although the duration of open-water events varied among stations. The following year, 2019, was another low sea ice year although the observed sea ice breakup resembled pre-2018 dates in DBO2 with some open water presence in March. Meanwhile at DBO1, 2019 sea ice breakup dates remained anomalously early and increases in daily sea ice concentration were less striking than in spring 2018.

Pheophytin proportions in high vs. low sea ice years were significantly different (Welch’s t-test, *p*<0.05) except for surface samples binned using the timing of sea ice breakup date (Table 2). The mean pheophytin proportions observed in July were consistently higher in high ice years than low ice years.

**Table 2:** Statistical summary for pheophytin proportions of total chlorophyll *a* + pheophytin measured in high vs. low sea ice station years for the DBO1 and DBO2 stations (n = 13) in the northern Bering Sea.

	Depth Integrated Pheophytin Proportions				Surface Pheophytin Proportions			
	Sea Ice Concentration		Sea Ice Breakup		Sea Ice Concentration		Sea Ice Breakup	
	Low Ice Years	High Ice Years	Low Ice Years	High Ice Years	Low Ice Years	High Ice Years	Low Ice Years	High Ice Years
N	20	52	46	26	20	51	45	26
Mean	0.35	0.42	0.38	0.45	0.25	0.34	0.30	0.35
SD	0.09	0.13	0.11	0.13	0.07	0.15	0.13	0.14
Min	0.18	0.11	0.11	0.26	0.15	0.14	0.14	0.15
Max	0.52	0.78	0.78	0.73	0.36	0.85	0.85	0.57
t-test	2.65		2.1		3.34		1.38	
*p*	0.01*		0.03*		0.001*		0.17	

N indicates the number of station year observations that were grouped in each high versus low year bin. The mean, standard deviation (SD), minimum, maximum are provided for the binned pheophytin proportions (Pheophytin/(Chlorophyll *a* + Pheophytin)) along with Welch’s t-test and *p*-value. *Bold values indicate significant differences at *p*<0.05.

**Fig 7:**
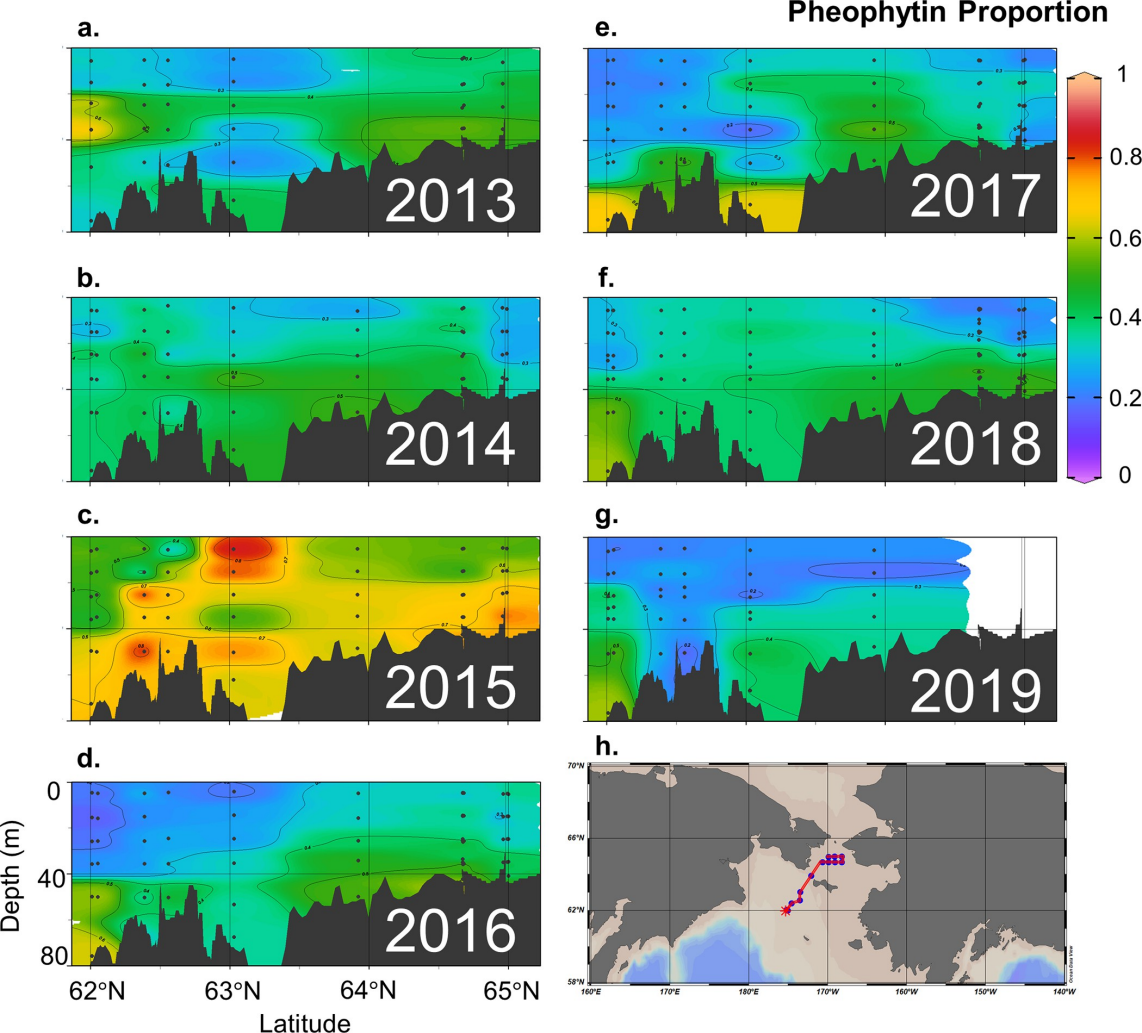
Transect views of pheophytin proportion based on combined chlorophyll *a* and pheophytin concentrations measured in units μg/L at each collected depth. The annual latitudinal transects show DBO1 stations on the left and DBO2 stations to the right. Note: cruise samples for most DBO2 stations were missing in 2019. Figure constructed using Ocean Data View (version 5.3; [46]).

**Fig 8:**
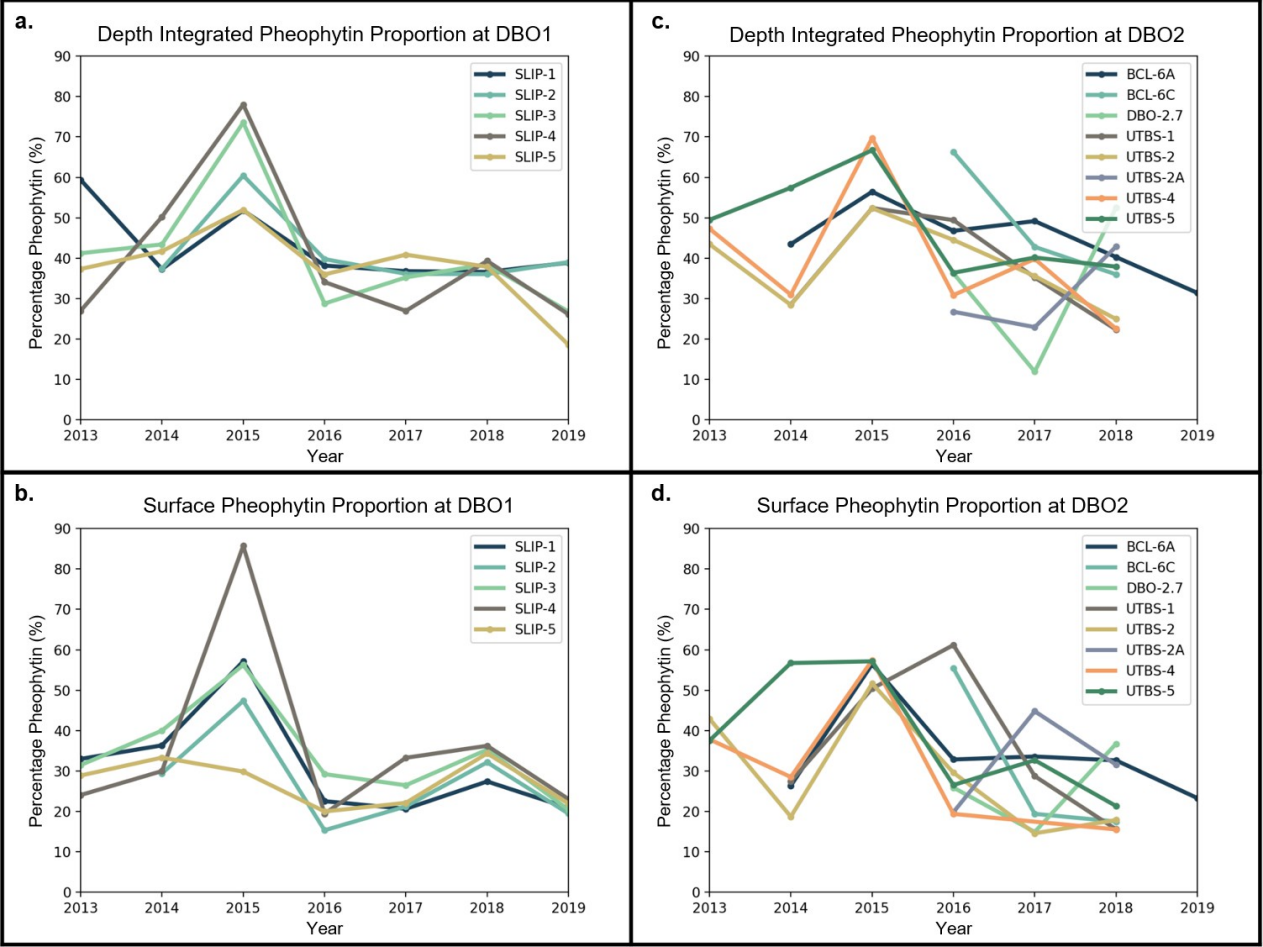
Pheophytin proportion time series. Depth integrated (a, c) and surface (b, d) pheophytin proportions at each station in DBO1 (a, b) and DBO2 (c, d) over time.

Pheophytin proportions throughout the water column showed interannual variability in July 2013–2019 (Figs 7 and 8). In 2015, no field EB signals were detected (Fig 3) as a sharp rise in pheophytin proportions was observed throughout the DBO1 and DBO2 stations (Figs 7 and 8), which corresponded to a high sea ice year at all stations based on sea ice concentration. Conversely, 2018 was classified as a low ice year but observations of July pheophytin proportions were not anomalously low relative to other years from 2013–2019 (Fig 8). However, there were more observations of field EB in 2018 (46%) than there were in previous high sea ice years such as 2013 (14%) and 2014 (30%) (Fig 3). In the following low ice year, 2019, 100% of the samples were classified as field EB though the number of samples collected was reduced in this particular year to N = 6 (Fig 3).

## Discussion

### In-situ and remotely sensed bloom stage model

The classification methodology used here sought to determine if pheophytin proportion (the ratio of pheophytin to combined pheophytin and chlorophyll *a*) from field measurements could be used to estimate bloom stage at the time of sampling based upon a satellite time series. To the best of our knowledge, this is the first application of this approach of basing remotely sensed classes on relation in time (growing or declining chlorophyll blooms) rather than direct snapshots of Earth’s surface to study the seasonality of phytoplankton growth stages. This application is based on the adaptation of remote sensing land cover classification techniques (e.g. [47]) to classify phytoplankton bloom stage at the time of field sampling.

Overall accuracy compares how each pixel is classified versus the confirmed surface cover conditions obtained from corresponding ground truth data [47]. While our methodology utilized chlorophyll *a* time series to estimate time-based classes, similar studies were unavailable for comparison. However, the overall accuracy of our classification model was 75%, which is consistent with previously published satellite-derived land cover classifications maps (e.g. [48]).

Examination of the misclassified station years revealed a general pattern. Each of the remotely sensed (RS) EB that were field-classified as PB contained large pheophytin proportions, ranging from 29–61%. These instances occurred as a second bloom following a recently ended bloom (over 2–33 day intervals). Therefore, the growing second bloom observed by satellite could not be distinguished in field samples due to elevated levels of pheophytin that remained in the water column from the preceding bloom. When considering the exact station matchups per year (Table 1), the pheophytin proportion model was successful at classifying satellite-derived PB in all cases. Despite the success in matching specific PB cases, inconsistencies (considered here as greater than three observations) between field and RS PB classes appeared in 6 out of the 7 years evaluated in the aggregated comparisons (Fig 3). However, these years also each coincided with 4–6 missing RS observations (labelled as RS NaN), which made up 16–27% of the total observations in any given year. Therefore, it is likely that the RS NaN would have contributed to the PB category had RS observations not been limited. Additionally, RS contained a “No Bloom” class which could not be differentiated using the pheophytin proportion index, which was a limitation between the field and RS classification matches.

Another limitation existed in using satellite observations as a reference because these data are also a proxy for surface level chlorophyll *a* biomass, and do not necessarily incorporate a confirming at-sea measurement. Furthermore, in order to take advantage of several years of useable data, our remote sensing time series included an expansion of the minimum observations criteria to once in at least every 25 days rather than once in 20 days that was used in prior work in this region [14, 31]. However, our criteria were adequate to resolve phenological patterns.

The frequency of EB were consistently identified in both the field and remotely sensed datasets year-to-year (r^2^ = 0.94, *p*< 0.01). EB were more prevalent in later years (2016–2019), yet the variability was reflected well in both models. Considering the satellite-derived bloom stages as the reference for individual matchups (Table 1), the pheophytin model performed well in classifying bloom stages with a success rate of 70% for EB. Overall, our model supports the conclusion that a pheophytin proportion of 28% or less based on the total concentration of chlorophyll *a* and pheophytin depicts pre-peak growth according to the accompanying satellite time series (Table 1). This model is novel in its ability to resolve phytoplankton growth versus deterioration within bloom cycles. Applying this model to other Bering Sea datasets would be beneficial to confirm the consistency of this 28% threshold.

Previous applications of using pheophytin proportions to estimate in-situ bloom stage are limited. Roy [49] routinely measured chlorophyll and associated pigments throughout a bloom progression in Bedford Basin, Canada and found a distinct difference in pheophytin proportion based on early and late bloom stages. Despite geographical differences, the dominant phytoplankton taxa studied by Roy [49] were diatoms, which is similarly dominant in the northern Bering Sea [50, 51]. Chlorophyll *a* represented 60% of the total pigment in the early bloom stage [49] while our model predicted a 72% representative threshold. However, Roy [49] measured chlorophyll *c* pigment separately, and those concentrations combined with chlorophyll *a* accounted for 70–75% of the early bloom signal at 5 m depth (Fig 5 in Roy [49]). Unfortunately, our observations did not differentiate additional chlorophyll accessory pigments. During the late bloom period, Roy [49] observed a distinct increase in surface pheophytin. Similarly, in the Laptev Sea another study observed high pheophytin concentrations compared to chlorophyll late in the season (September) [24]. Additionally, Sathish et al. [52] found seasonal trends in pheophytin concentrations after repeated sampling in various marine ports on the Indian subcontinent. These findings support the suitability of pheophytin proportions as an index for bloom stage.

### Pheophytin proportions in the northern Bering Sea

On the Chukchi Shelf, Goñi et al. [53] found bottom waters with high (>0.4) pheophytin proportions (reported as pheophytin/(chlorophyll + pheophytin) ratios) indicative of phytoplankton detritus in August and September. Interestingly, in July and August, McTigue et al. [54] found the ratio of chlorophyll to total pheopigments (the sum of pheophytin *a*, pheophorbide *a*, and pyropheophorbide *a*) to be > 1 at the majority of their Chukchi Shelf stations for surface sediment concentrations, indicating viable cells. Goñi et al. [53] reported similar results and found that surface sediments that received fresh inputs of phytoplankton cells had low (< 0.2) pheophytin proportions whereas locations with increased microbial and herbivorous grazing displayed higher (>0.2) pheophytin proportions. While we did not measure sediment pigment concentrations, the pheophytin proportions measured in the northern Bering Sea bottom waters were consistently greater than 0.2 and were commonly greater than 0.4 (Fig 7). While these proportions are not consistent with the sediment processes described by McTigue et al. [54], the July northern Bering Sea bottom water pheophytin proportions were on par with late summer Chukchi Sea samples reported by Goñi et al. [53].

### Sea ice and pheophytin proportion dynamics

Pheophytin proportions differed between high and low ice years for samples collected at the surface as well as depth integrated inventories. These data provided consistent evidence of a connection between the timing of sea ice dissolution and pheophytin proportions (Table 2). High sea ice years were associated with a larger mean pheophytin proportion than was found in low ice years in the northern Bering Sea (Welch’s t-test, *p*<0.05) in three of four analyses, although surface samples grouped by sea ice breakup of low and high years were not significant. The surface samples analyzed according to sea ice breakup had the smallest difference in mean (0.05) and nearly exact standard deviation (difference of 0.01) between the low and high ice year groups, which likely resulted in the insignificant distinction among them. Still, in most of the cases high versus low sea ice years produced statistically different outcomes for the observed pheophytin proportions.

While distinct outcomes were found between high versus low sea ice years when aggregating DBO1 and DBO2 stations through time, the relationship was less obvious in individual years. Annually, the pheophytin proportion throughout the water column did not exhibit a clear distinction between the high and low years (Figs 7 and 8). Individual years and stations had high variability and there is a need for a longer time series at individual stations. Interannual variability and exceptions of years with high (low) sea ice producing low (high) pheophytin proportions suggest sea ice is not the sole driver of bloom stage in mid-summer, despite statistical indications that sea ice influences chlorophyll *a* and pheophytin proportions (Table 2). Factors such as stratification and nutrient availability are major drivers of phytoplankton growth and taxonomic changes. These mid-summer conditions likely influenced July chlorophyll *a* concentrations.

Results of our classification model indicate it is practical to define bloom stage based on pheophytin proportion relative to chlorophyll *a*. Lower proportions of pheophytin in low sea ice years suggests an early bloom signal will be present in mid-summer compared to a more mature (higher pheophytin proportion) bloom stage that is typical in high sea ice years. Prior work on these dynamics, e.g. the Oscillating Control Hypothesis [55] projected that late ice retreat leads to an early, ice-associated bloom, but limited ice or an early ice retreat leads to a later open-water spring bloom in May or June in warm water. This hypothesis assumes that the timing of spring primary production is primarily determined by the timing of ice retreat. Sea ice cover aids phytoplankton growth by providing freshwater stratification from sea ice melt that provides a stable water column to support phytoplankton growth in the euphotic zone, and by mixing nutrients into the upper water column during winter brine rejection as sea ice forms [56]. Although our samples were obtained in July, months after direct interaction between sea ice and the spring bloom, we were able to distinguish a shift in the phase of phytoplankton phenology based on pheophytin proportions. Following from the Oscillating Control Hypothesis, our results indicate that earlier sea ice breakup and low springtime sea ice concentrations that alter spring bloom timing have consequences for chlorophyll *a* biomass extending into mid-summer. In short, the detection of this phase shift with increased observations of EB rather than PB conditions in low ice years suggests that changes in timing of the spring bloom further alters mid-summer production.

Other observations simultaneous with our study include the M5 mooring southeast of St. Matthew Island in the Bering Sea, where observations were made of low chlorophyll *a* concentrations within a late spring bloom (with onset on approximately June 12) in 2018 owing to the lack of fresh water input from melting sea ice resulting in weak stratification [8]. Kikuchi et al. [57] also observed delays in the spring bloom at their mooring sites north and south of the Bering Strait in 2018 compared to 2017, and attributed the differences to varying sea ice concentrations. Observations and modelling [15] of later spring blooms that follow spring seasons with little or no sea ice could conceivably lead to delayed phytoplankton growth in later months. Though other environmental variables clearly influence mid-summer productivity, this study demonstrates that sea ice status during the prior spring, including concentrations and breakup date are contributing drivers.

The absence of winter sea ice in the Bering Sea in 2018 could become typical by the 2040s [2]. It is possible that these anomalous events indicate the beginning of a sea ice regime change that could reflect ecosystem restructuring [58]. Based on the 2013–2019 July observations, continued sea ice decline may shift mid-summer productivity to an earlier bloom signal than previously observed in the northern Bering Sea. Delayed spring blooms that lead to delayed summer bloom pulses could change the timing of food availability for higher trophic levels. While the spring bloom is considered the most critical period for biological production, there is evidence in the southeastern Bering Sea that summer production is also a crucial energy source for zooplankton and contributes to the survival of age-0 walleye pollock [59]. Long term consequences of a shift in mid-summer production is uncertain. Ice cover in the 2019/2020 winter was more extensive, providing uncertainty for whether 2017/2018 sea ice conditions represent an anomaly or the beginning stages of a new regime state [60]. Still, major environmental changes in this region are expected to continue and will require expanded efforts to include both remotely sensed and field observations to resolve physical and ecological consequences of climate change.

## Conclusion

The classification model presented is shown to be able to resolve phytoplankton growth stages within bloom cycles from in-situ discrete water samples. An optimized model for northern Bering Sea field samples produced a threshold of a proportion of 28% pheophytin in the overall pheophytin + chlorophyll *a* pigment inventory to distinguish growing blooms from post-peak bloom stages. Our study found that samples that consisted of less than 28% pheophytin proportion represented growing blooms while proportions higher than 28% indicated past peak or senescent blooms according to remotely sensed chlorophyll *a* phenology. Although additional studies are needed to confirm this model with other northern Bering Sea datasets, our results suggest that pheophytin proportions can be used as a proxy for bloom stage as confirmed by satellite sensed phenology.

Earlier sea ice breakup dates and reduced springtime sea ice concentrations led to a later spring bloom and subsequently an increase in early bloom stages (as opposed to matured blooms) being observed in mid-summer. The difference of pheophytin proportions from July cruise samples between high and low sea ice years was significant (*p*<0.05) for depth-integrated pheophytin. Surface pheophytin proportions were divided between two distinctive outcomes with high confidence (*p*<0.001) for March–May sea ice concentration. The increase in mid-summer EB signals observed in low sea ice years represents a shift from typical annual growth cycles. The Oscillating Control Hypothesis [55] describes the mechanisms driving delayed spring blooms following early sea ice retreat. Therefore, it is likely that changes to the timing of spring phytoplankton blooms owing to decreased sea ice can have pronounced effects on mid-summer production, though the influence of drivers such as immediate nutrient availability and stratification may prevail in individual years.

## Supporting information

S1 Appendix Sea ice breakup change point analysis. (DOCX)

S1 Table Day of sea ice breakup for each station per year. The sea ice breakup dates are expressed in Day of Year (DOY). Italicized dates are based on 2017 point location as a baseline because in situ samples were not gathered at the corresponding station.
(DOCX)

S2 Table Time of sampling phytoplankton life stage investigated with surface field and remotely sensed chlorophyll. Sampled surface chlorophyll, pheophytin, the associated portion of pheophytin relative to combined pheophytin and chlorophyll concentrations, and the classified life stage of the bloom according to satellite remotely sensed phenology at the DBO1 (top) and DBO2 (bottom) stations. For the remotely sensed life stage categories, NaN = Not enough cloud-free satellite observations available to resolve annual phenology, EB = Early Bloom, PB = Post Bloom, NB = No Bloom. Surface chlorophyll and pheophytin are in units μg/L.
(DOCX)

S3 Table Sea ice breakup date changepoint analysis. The sea ice breakup dates changepoint analysis results to determine the day of year (DOY) threshold to distinguish high versus low sea ice years within our study duration per station. These were derived using the methodology described in S1 Appendix.
(DOCX)

S4 Table Sea ice high and low years and depth-integrated pheophytin proportions. High and low sea ice years were distinguished using DOY thresholds determined per station based on sea ice breakup date (SIB) and by sea ice concentration (SIC) relative March–May coverage at each station during the 2013–2019 time period.
(DOCX)
