# Supplementary material for: Phytoplankton bloom stages estimated from chlorophyll pigment proportions suggest delayed summer production in low sea ice years in the northern Bering Sea
Source: PLoS One. 2022 Jul 8;17(7):e0267586. doi: 10.1371/journal.pone.0267586 (PMC9269360; doi:10.1371/journal.pone.0267586)
Supplement: S1 Appendix — (DOCX) [file pone.0267586.s001.docx]

**S1 Appendix. Sea ice breakup change point analysis.**

Change point analysis was conducted to determine a date threshold to classify high versus low ice years according to sea ice breakup date per station over the years considered (2013-2019) using the ‘changepoint’ package in R (R Core Development Team). The package calculates the optimal positioning of changepoints for data. Within this package, the “cpt.mean” function was used to find changes in mean sea ice breakup date using the “AMOC” method which returns a single changepoint. The duration of the time series for the change point analysis was constrained to 2013-2019 to determine DOY thresholds of high or low ice years specific to the analysis of each station. Calculation of the DOY threshold for distinguishing high or low ice years were done via Equation 1 for each station individually in DBO1&2. Years were categorized as low or high sea ice years based on if the sea ice breakup occurred before or after the DOY threshold, respectively.


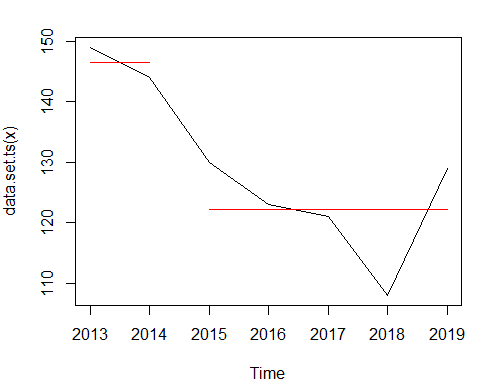


DOY mean_1_

DOY mean_2_

1.

Threshold DOY for high SIB

or low SIB categorization

= (DOYmean_1_ – DOYmean_2_)(1/2) + DOYmean_2_
