# Supplementary material for: Phytoplankton bloom stages estimated from chlorophyll pigment proportions suggest delayed summer production in low sea ice years in the northern Bering Sea
Source: PLoS One. 2022 Jul 8;17(7):e0267586. doi: 10.1371/journal.pone.0267586 (PMC9269360; doi:10.1371/journal.pone.0267586)
Supplement: S1 Table — The sea ice breakup dates are expressed in Day of Year (DOY). Italicized dates are based on 2017 point location as a baseline because in situ samples were not gathered at the corresponding station. (DOCX) [file pone.0267586.s002.docx]

**S1 Table. Day of sea ice breakup for each station per year.**

| **Transect** | **Station** | **2013** | **2014** | **2015** | **2016** | **2017** | **2018** | **2019** |
| --- | --- | --- | --- | --- | --- | --- | --- | --- |
| DBO1 | SLIP-1 | 148 | 131 | 117 | 113 | 121 | - | 85 |
|  | SLIP-2 | 148 | 131 | 117 | 113 | 121 | - | 85 |
|  | SLIP-3 | 150 | 113 | 117 | 112 | 114 | 79 | 87 |
|  | SLIP-4 | 163 | 112 | 117 | 111 | 111 | 82 | 88 |
|  | SLIP-5 | 159 | 112 | 116 | 111 | 113 | 81 | 87 |
| DBO2 | BCL-6A | *154* | 152 | 117 | 131 | 102 | 84 | 118 |
|  | BCL-6C | *136* | *140* | *127* | 122 | 105 | 90 | 118 |
|  | UTBS-5 | 137 | 140 | 129 | 125 | 108 | 90 | 125 |
|  | UTBS-4 | 137 | 140 | 129 | 125 | 106 | 90 | 119 |
|  | UTBS-2 | 149 | 143 | 129 | 124 | 115 | 90 | 130 |
|  | UTBS-2A | *147* | *143* | *141* | 119 | 125 | 107 | 92 |
|  | UTBS-1 | 149 | 144 | 130 | 123 | 121 | 108 | 129 |
|  | DBO2.7 | *148* | *145* | *142* | 119 | 128 | 112 | *93* |

The sea ice breakup dates are expressed in Day of Year (DOY). Italicized dates are based on 2017 point location as a baseline because in situ samples were not gathered at the corresponding station.
