# Supplementary material for: Phytoplankton bloom stages estimated from chlorophyll pigment proportions suggest delayed summer production in low sea ice years in the northern Bering Sea
Source: PLoS One. 2022 Jul 8;17(7):e0267586. doi: 10.1371/journal.pone.0267586 (PMC9269360; doi:10.1371/journal.pone.0267586)
Supplement: S2 Table — Sampled surface chlorophyll, pheophytin, the associated portion of pheophytin relative to combined pheophytin and chlorophyll concentrations, and the classified life stage of the bloom according to satellite remotely sensed phenology at the DBO1 (top) and DBO2 (bottom) stations. For the remotely sensed life stage categories, NaN = Not enough cloud-free satellite observations available to resolve annual phenology, EB = Early Bloom, PB = Post Bloom, NB = No Bloom. Surface chlorophyll and pheophytin are in units μg/L. (DOCX) [file pone.0267586.s003.docx]

**S2 Table.** **Time of sampling phytoplankton life stage investigated with surface field and remotely sensed chlorophyll.**

| DBO1 | Station | Sample DOY | Surface Pheo | Surface Chl | Pheo Portion | RS Category |
| --- | --- | --- | --- | --- | --- | --- |
| 2013 | SLIP-1 | 195 | 0.07 | 0.13 | 0.33 | NaN |
|  | SLIP-3 | 195 | 0.05 | 0.11 | 0.31 | NaN |
|  | SLIP-5 | 195 | 0.04 | 0.10 | 0.29 | NaN |
|  | SLIP-4 | 195 | 0.06 | 0.18 | 0.24 | NaN |
| 2014 | SLIP-1 | 195 | 0.04 | 0.07 | 0.36 | NB |
|  | SLIP-2 | 195 | 0.05 | 0.12 | 0.29 | NB |
|  | SLIP-3 | 195 | 0.04 | 0.06 | 0.40 | NaN |
|  | SLIP-5 | 196 | 0.02 | 0.04 | 0.33 | NaN |
|  | SLIP-4 | 196 | 0.03 | 0.07 | 0.30 | NaN |
| 2015 | SLIP-1 | 195 | 0.08 | 0.06 | 0.57 | NaN |
|  | SLIP-2 | 195 | 0.09 | 0.10 | 0.47 | NaN |
|  | SLIP-3 | 195 | 0.09 | 0.07 | 0.56 | NaN |
|  | SLIP-5 | 196 | 0.66 | 1.55 | 0.30 | NB |
|  | SLIP-4 | 196 | 0.06 | 0.01 | 0.86 | NaN |
| 2016 | SLIP-1 | 194 | 0.05 | 0.16 | 0.23 | EB |
|  | SLIP-2 | 195 | 0.03 | 0.17 | 0.15 | NaN |
|  | SLIP-3 | 195 | 0.03 | 0.08 | 0.29 | NB |
|  | SLIP-5 | 195 | 0.04 | 0.14 | 0.20 | NB |
|  | SLIP-4 | 195 | 0.02 | 0.09 | 0.19 | NaN |
| 2017 | SLIP-1 | 195 | 0.04 | 0.16 | 0.21 | EB |
|  | SLIP-2 | 195 | 0.07 | 0.25 | 0.21 | EB |
|  | SLIP-3 | 195 | 0.08 | 0.22 | 0.26 | NaN |
|  | SLIP-5 | 196 | 0.06 | 0.21 | 0.22 | NB |
|  | SLIP-4 | 196 | 0.04 | 0.08 | 0.33 | NaN |
| 2018 | SLIP-1 | 197 | 0.10 | 0.27 | 0.27 | NaN |
|  | SLIP-2 | 197 | 0.14 | 0.30 | 0.32 | NaN |
|  | SLIP-3 | 197 | 0.12 | 0.21 | 0.35 | NaN |
|  | SLIP-5 | 198 | 0.19 | 0.36 | 0.34 | NaN |
|  | SLIP-4 | 198 | 0.21 | 0.37 | 0.36 | NB |
| 2019 | SLIP-1 | 195 | 0.11 | 0.41 | 0.21 | EB |
|  | SLIP-2 | 195 | 0.20 | 0.82 | 0.19 | EB |
|  | SLIP-3 | 195 | 0.09 | 0.33 | 0.20 | NaN |
|  | SLIP-5 | 195 | 0.10 | 0.36 | 0.22 | NB |
|  | SLIP-4 | 195 | 0.06 | 0.20 | 0.23 | NB |

| DBO2 | Station | Sample DOY | Surface Pheo | Surface Chl | Pheo Portion | RS Category |
| --- | --- | --- | --- | --- | --- | --- |
| 2013 | UTBS-5 | 196 | 0.08 | 0.13 | 0.38 | NB |
|  | UTBS-4 | 196 | 0.31 | 0.51 | 0.38 | PB |
|  | UTBS-2 | 196 | 0.27 | 0.36 | 0.43 | EB |
| 2014 | BCL-6A | 196 | 0.19 | 0.53 | 0.26 | EB |
|  | UTBS-5 | 197 | 0.80 | 0.61 | 0.57 | PB |
|  | UTBS-2 | 197 | 0.29 | 1.26 | 0.19 | NaN |
|  | UTBS-4 | 197 | 0.81 | 2.03 | 0.29 | PB |
|  | UTBS-1 | 197 | 0.54 | 1.43 | 0.27 | EB |
| 2015 | BCL-6A | 196 | 0.57 | 0.44 | 0.56 | PB |
|  | UTBS-5 | 196 | 0.76 | 0.57 | 0.57 | EB |
|  | UTBS-2 | 197 | 2.35 | 2.20 | 0.52 | NB |
|  | UTBS-4 | 197 | 1.24 | 0.92 | 0.57 | NaN |
|  | UTBS-1 | 197 | 1.21 | 1.19 | 0.50 | NaN |
| 2016 | BCL-6A | 195 | 0.10 | 0.20 | 0.33 | NB |
|  | BCL-6C | 196 | 0.30 | 0.24 | 0.55 | EB |
|  | UTBS-5 | 196 | 0.12 | 0.33 | 0.27 | EB |
|  | UTBS-2 | 196 | 0.13 | 0.31 | 0.30 | EB |
|  | UTBS-2A | 196 | 0.07 | 0.30 | 0.20 | NaN |
|  | DBO2.7 | 196 | 0.13 | 0.37 | 0.26 | NaN |
|  | UTBS-1 | 196 | 1.21 | 0.77 | 0.61 | EB |
|  | UTBS-4 | 197 | 0.07 | 0.30 | 0.19 | EB |
| 2017 | BCL-6A | 196 | 0.11 | 0.21 | 0.34 | EB |
|  | BCL-6C | 196 | 0.19 | 0.81 | 0.19 | NaN |
|  | UTBS-5 | 197 | 0.24 | 0.49 | 0.33 | EB |
|  | UTBS-2 | 197 | 0.24 | 1.43 | 0.15 | EB |
|  | UTBS-2A | 197 | 0.19 | 0.24 | 0.45 | NaN |
|  | DBO2.7 | 197 | 0.36 | 2.05 | 0.15 | NaN |
|  | UTBS-1 | 197 | 0.25 | 0.63 | 0.29 | PB |
| 2018 | BCL-6A | 198 | 0.12 | 0.25 | 0.33 | NB |
|  | BCL-6C | 199 | 0.64 | 3.03 | 0.18 | EB |
|  | UTBS-5 | 199 | 0.38 | 1.41 | 0.21 | EB |
|  | UTBS-2 | 199 | 1.40 | 6.44 | 0.18 | EB |
|  | UTBS-2A | 199 | 0.39 | 0.85 | 0.32 | NaN |
|  | DBO2.7 | 199 | 0.30 | 0.51 | 0.37 | NaN |
|  | UTBS-1 | 199 | 0.94 | 5.08 | 0.16 | EB |
|  | UTBS-4 | 199 | 0.97 | 5.29 | 0.16 | EB |
| 2019 | BCL-6A | 196 | 0.21 | 0.69 | 0.23 | EB |

Sampled surface chlorophyll, pheophytin, the associated portion of pheophytin relative to combined pheophytin and chlorophyll concentrations, and the classified life stage of the bloom according to satellite remotely sensed phenology at the DBO1 (top) and DBO2 (bottom) stations. For the remotely sensed life stage categories, NaN = Not enough cloud-free satellite observations available to resolve annual phenology, EB = Early Bloom, PB = Post Bloom, NB = No Bloom. Surface chlorophyll and pheophytin are in units μg/L.
