# Supplementary material for: Phytoplankton bloom stages estimated from chlorophyll pigment proportions suggest delayed summer production in low sea ice years in the northern Bering Sea
Source: PLoS One. 2022 Jul 8;17(7):e0267586. doi: 10.1371/journal.pone.0267586 (PMC9269360; doi:10.1371/journal.pone.0267586)
Supplement: S3 Table — The sea ice breakup dates changepoint analysis results to determine the day of year (DOY) threshold to distinguish high versus low sea ice years within our study duration per station. These were derived using the methodology described in S1 Appendix. (DOCX) [file pone.0267586.s004.docx]

**S3 Table. Sea ice breakup date changepoint analysis.**

The sea ice breakup dates changepoint analysis results to determine the day of year (DOY) threshold to distinguish high versus low sea ice years within our study duration per station. These were derived using the methodology described in S1 Appendix.
