# Supplementary material for: Phytoplankton bloom stages estimated from chlorophyll pigment proportions suggest delayed summer production in low sea ice years in the northern Bering Sea
Source: PLoS One. 2022 Jul 8;17(7):e0267586. doi: 10.1371/journal.pone.0267586 (PMC9269360; doi:10.1371/journal.pone.0267586)
Supplement: S4 Table — High and low sea ice years were distinguished using DOY thresholds determined per station based on sea ice breakup date (SIB) and by sea ice concentration (SIC) relative March–May coverage at each station during the 2013–2019 time period. (DOCX) [file pone.0267586.s005.docx]

**S4 Table. Sea ice high and low years and depth-integrated pheophytin proportions.**

High and low sea ice years were distinguished using DOY thresholds determined per station based on sea ice breakup date (SIB) and by sea ice concentration (SIC) relative March–May coverage at each station during the 2013-2019 time period.
